# Nutritional composition and antidiabetic effect of germinated endosperm (*Borassus flabellifer*), tuber (*Amorphophallus paeoniifolius*) and their combined impact on rats

**DOI:** 10.1016/j.bbrep.2021.100917

**Published:** 2021-01-20

**Authors:** Shaikh Shahinur Rahman, Hussain Mohammad Salauddin, Mahfuzur Rahman, Mir Mohammad Muhsin, Shakh MA Rouf

**Affiliations:** a Department of Applied Nutrition and Food Technology, Islamic University, Kushtia, Bangladesh; b Bangladesh Agricultural Research Council (BARC), Farmgate, Dhaka, Bangladesh

**Keywords:** Antidiabetic, Nutrients, Elephant foot yam, Sugar palm, Germinated endosperm, Rats

## Abstract

Diabetic patients usually avoid germinated endosperm of sugar palm (GESP) and elephant foot yam tuber (EFYT), fearing that these may further deteriorate existing hyperglycemia. In the present study, this suspicion was investigated by analyzing the nutrients and following the animal experiments by supplementary feeding powder of GESP, EFYT, and their mixture in addition to the regular diet for the six consecutive weeks. Next three weeks, the powder was withdrawn, and fasting blood glucose (FBG) levels were recorded from the beginning. The results clearly showed that these foodstuffs significantly (*P* < 0.001) reduced FBG levels of alloxan-induced diabetic rats. The mixture of GESP & EFYT showed the maximum antidiabetic effects followed by GESP and EFYT, respectively. GESP, as well as the mixture, returned the FBG levels of diabetic rats within the normal range by the end of the 6th week, even after withdrawing the powder, but not by the EFYT. These results suggested that the foodstuffs may restore the damaged pancreatic β-cell functions by the end of the 6th week. Nutrient contents like fiber, zinc, as well as antidiabetogenic phytochemicals present in these foodstuffs, could perform these functions.

## Highlights

• The germinated endosperm of sugar palm (*Borassus flabellifer)* has the highest antidiabetogenic phytochemicals.
• The combined impact of germinated endosperm and tuber had a significant level of antidiabetic action.
• Both foodstuffs were rich with a complex carbohydrate, fiber, zinc, minerals, as well as phytochemicals.

## 1 Introduction

Diabetes mellitus (DM) is a metabolic disorder characterized by persistent hyperglycemia [1] and is caused by insulin resistance, defective insulin secretion, or both genetic and environmental factors [2]. From ancient, many synthetic drugs (viz. recombinant insulin & oral hypoglycemic agents) and herbal products were used to maintain a normoglycemic condition [3]. World Health Organization (WHO) emphasized and recommended traditional remedies to control DM due to safe and efficiently available at low cost [4].

Elephant foot yam (*Amorphophallus paeoniifolius*) tuber (EFYT) is mainly used as a vegetable in various cuisines and is also considered a major ingredient in Ayurvedic prescriptions [5]. It is widely used by different tribes to treat many chronic, infectious, and fatal diseases viz. antiinflammatory, anti haemorrhoidal, hepatoprotective, stomachic, analgesic, cytotoxic, antihelminthic, antifungal, antibacterial, antiprotease, and CNS depressant activities [6,7]. Moreover, patients with diabetes avoid EFYT due to having fear about this tuber that it may raise serum glucose level [8]. But there is no scientific evidence favoring such a misconception.

On the other hand, germinated endosperm of sugar palm (GESP) fruit (scientifically known as *Borassus flabellifer*) is also popularly consumed as a raw or vegetable throughout the Indian subcontinent and Southeast Asia. But many rural and urban diabetic patients in Bangladesh avoid this GESP due to having their common belief on the negative impact on diabetes. However, no research data was found either to support or to reject these existing taboos. Hence, the present study was designed to investigate the effect of GESP, EFYT, and their combination on the fasting blood glucose (FBG) level in alloxan-induced diabetic rats.

## 2 Materials and methods

### 2.1 Sample collection

EFYT (*A. paeoniifolius*) and GESP (*B. flabellifer*) were collected from the local farmer of the South-West region, Bangladesh, from October to December. These samples were identified by the expert group of the Faculty of Biological Science, Islamic University, Bangladesh with ref no. FBS/ERC/2019.

### 2.2 Sample preparation

Samples were cleaned, pilled, and chopped into small pieces followed by dried in a thermostatically controlled oven at 50 °C for 24 h. The dried samples were converted into powder using a grinder and test sieve, no. 80 mesh. The powder was then packaged in lidded polyethylene containers until nutritional analysis and animal experiments.

### 2.3 Extraction

About 50 gm of the dried powder of EFYT and GESP were soaked in 600 ml of a chloroform-methanol mixture (2:1v/v) at room temperature for 48 h and then filtered with Whatman No. 42 (125 mm) filter paper to collect the supernatant. The supernatant was then poured into a round-bottomed flask at low pressure (60 rpm at 37 °C) to remove the excess methanol via evaporation. However, three layers (a clear lower layer of chloroform contained all the lipids, a dark-brown colored aqueous layer of methanol with all water-soluble material, and a thick pasty interface) were seen when the resulting solution was subjected to centrifugation. The concentrated methanol extract was suspended in the distilled water then extracted with n-hexane, chloroform, ethyl acetate, and n-butanol sequentially. The mixtures were shaken vigorously and were made to stand for some time for proper separation. The extracts were stored at 4 °C in airtight bottles and were qualitatively tested for the presence of various phytocompounds.

### 2.4 Nutritional analysis

The nutrient contents of EFYT and GESP were estimated according to the standard analytical methods [9]. The carbohydrate content was determined by the calculated difference method. The energy value was determined by multiplying the proportion of protein, fat, and carbohydrate by their respective energy values and taking the sum of the products. Sodium, potassium, calcium, and phosphorus contents were determined by a flame photometric method using a systronics type 130 flame photometer [10]. All chemicals used in this study were of analytical reagent grade (Sigma Aldrich), and the working solutions were prepared by dilution of the appropriate amounts of each mineral (100 ppm calcium, 200 ppm potassium, and 200 ppm sodium) from the standard stock solutions (1000 μg/ml). In this method, 1 g of sample was mixed with 20 ml of a di-acid mixture (4 HNO3:1 HClO4) and taken into a 100 ml conical flask. Then, the sample was kept overnight and digested at a low temperature on the hot plate. The digestion was continued until the liquid turns into colorless [11].

Iron, copper, zinc, magnesium, and manganese were determined by the Flame Atomic Absorption Spectrophotometer (wavelength 248.3–327 nm). About 5–15 g samples were dried in an air oven at 105 °C for 3 h and then burned in a muffle furnace at 550 °C to obtain greyish ash. The ash was taken in a volumetric flask and mixed with a concentrated hydrochloric acid (50 ml). Ferric nitrate solution was used as standard, and concentrations of iron in the experimental solutions were calculated from the standard curve [12].

Vitamin C was determined by the Indophenol method as per the procedure as outlined by the Food Analysis Laboratory Manual [13]. Total carotenoid was determined using the method described by Speek et al. [14]. The amount of soluble protein and non-protein nitrogen was calculated by determining soluble nitrogen by the Kjeldahl method [15].

### 2.5 Phytochemical screening

Qualitative phytochemicals analysis of EFYT and GESP extracts was performed by following the standard procedures described by Harborne [16] and Lay [17].

### 2.6 Animals

Healthy Long-Evans male rats (90–150 g) were kept in the standard laboratory conditions, temperature (24 °C±2), and humidity 45 ± 5% with 12h day: 12h night cycle. Rats were divided randomly into eight groups, and each group consisted of six rats. Among them, two groups were nondiabetic control (NC) and diabetic control (DC). At the same time, the other six groups were experimental groups: N1 (nondiabetic GESP), N2 (nondiabetic EFYT), N3 (nondiabetic GESP + EFYT), D1 (diabetic GESP), D2 (diabetic EFYT), and D3 (diabetic GESP + EFYT).

### 2.7 Diet and feeding procedure of the rats

Animals were fed a standard diet proposed by Hafizur et al. [18] that made by mixing: wheat flour (30%), wheat bran (21%), rice polish (20%), fish-meal (10%), oilseed cake (10%), molasses (5%), soybean oil (2%), common salt (1.5%) and multivitamins (0.5%). According to the National Research Council [19], a dietary intake of 15 g/rat/day was given to the rats. The investigation of this study was continued for nine consecutive weeks. Among these experimental periods, dry powder (500 mg/kg.bd.wt./rat) of GESP, EFYT, and the mixture of GESP & EFYT (1:1) were orally fed (through feeding syringe) up to 6th weeks in addition to the regular diet. Next, three weeks of this study, the supplementary feeding diet was pulled out.

### 2.8 Induction of diabetes

All the chemicals and reagents were the analytical grade and purchased from Sigma-Aldrich (St. Louis, MO, USA). Alloxan monohydrate (stored at 4 °C) was dissolved in normal saline at room temperature, and intraperitoneal routes in overnight fasted rats injected 140 mg/kg body weight [1]. After 72 h, the fasting blood glucose (FBG) level was determined from the tail vein by using Glucosure strips from Apex Bio, Taiwan. Animals with FBG >250 mg/dl (>13.8 mmol/L) were considered diabetic and were included in this study.

### 2.9 Statistical analysis

Data were analyzed using SPSS software for windows version 11.5. All results were expressed as the mean ± SD (Standard Deviation). One-way analysis of variance (ANOVA) used and paired or unpaired *t*-test was done for multiple comparisons between groups. The values of p < 0.05 were considered statistically significant, and p < 0.001 were highly significant.

### 2.10 Ethical issues

This study was carried out following the ethical guidelines of the Institutional Animal Ethical Committee, Faculty of Biological Science, Islamic University, Kushtia, Bangladesh. The Institutional Review Board of the Islamic University approved this study.

## 3 Result

### 3.1 Quantitative analysis of nutrients

The nutrient contents of EFYT & GESP were considered on a dry basis and calculated per 100 g dry powder of each sample. The results obtained are presented in Table 1, and it is seen that the GESP had lower carbohydrate content (59.73%) than the EFYT (71.71%). In contrast, the crude fiber of GESP (5.15%) had remarkably higher (*P* < 0.001) than EFYT (1.67%). Both samples' protein content had almost a similar quantity (12.13–12.43%), but the GESP had significantly higher (around three times) crude fat and ash content than EFYT. The high ash content of GESP indicates that it can be a good source of dietary minerals. Besides this, GESP can help improve the digestive system because of the high fiber content. The calculated energy values of GESP and EFYT were 301.63 and 347.48 Kcal, respectively, for 100 g powder. However, mineral contents were higher in GESP, and zinc, copper, iron, sodium, and potassium were found significantly (*P* < 0.001) higher in the germinated endosperm compare to the EFYT (Table 1). On the other hand, the vitamin-C content of EFYT had four times more than GESP. Free fatty acid and non-protein nitrogen of both samples were in a negligible amount. The protein solubility of GESP was greater (93.70%) than EFYT (86.90%). Moreover, total carotenoids of GESP had significantly higher (*P* < 0.001) than that of EFYT (0.26%).

**Table 1:** Proximate nutrient analysis of EFYT and GESP (per 100 g dry basis).

Nutrient	GESP	EFYT
Dry matter (g)	87.60^b^ ± 0.6	89.97^a^±1.42
Moisture (g)	12.40^a^±0.16	10.03^b^ ± 0.01
Carbohydrate (g)	59.73^b^ ± 0.8	71.71^a^**±1.74
Crude fiber (g)	5.15^a^**±0.37	1.67^b^ ± 0.12
Crude protein (g)	12.13^b^ ± 0.2	12.43^a^±0.65
Crude fat (g)	0.77^a^**±0.03	0.26^b^ ± 0.02
Ash (g)	9.40^a^**±0.22	3.90^b^ ± 0.2
Energy value (Kcal)	301.63^b^ ± 0.95	347.48^a^±3.15
Calcium (mg)	0.17^a^±0.01	0.09^b^ ± 0.01
Phosphorus (mg)	0.26^a^±0.01	0.04^b^ ± 0.01
Iron (mg)	0.56^a^**±0.01	0.07^b^ ± 0.01
Copper (mg)	0.78^a^**±0.14	0.01^b^ ± 0.01
Zinc (mg)	0.87^a^**±0.11	0.02^b^ ± 0.01
Magnesium (mg)	0.08^a^±0.01	0.05^b^ ± 0.04
Manganese (mg)	0.09^a^±0.02	0.01^b^ ± 0.01
Sodium (mg)	52.35^a^**±1.61	0.06^b^ ± 0.02
Potassium (mg)	154.91^a^**±1.2	0.05^b^ ± 0.03
Vitamin-C (mg)	1.81^b^ ± 0.09	5.57^a^**±0.41
Total carotenoids (mg)	3.68^a^**±0.15	0.26^b^ ± 0.04
Non protein nitrogen	Trace	Trace
Protein solubility (mg/ml)	93.70^a^±0.9	86.90^b^ ± 1.21
Free fatty acid (FFA)	Trace	0.09^a^±0.01

Values are means of triplicates ± standard deviation. Superscript ‘a’ and ‘b’ in a row indicates higher & lower values respectively.

^a^Indicates significantly different (*P* < 0.05) and.

^b^Indicates highly significant (*P* < 0.001) as determined by Duncan's multiple range test.

### 3.2 Qualitative analysis of phytochemicals

Twenty-five phytochemicals believed to have antidiabetic roles either directly or indirectly were also tested (qualitatively) for their presence in the GESP and EFYT. Among them, twenty-three phytochemicals were found in the GESP, and only seventeen phytochemicals were present in the EFYT, shown in Table 2. Phytochemicals viz. alkaloid, albuminoids, anthracene, betulinic acid, flavonoid, free anthraquinone, glucomannan, gums, lupeol, quercetin, reducing compounds, β-sitosterol, steroid, sterols, stigmasterols, and terpenoid were present in both samples. Phlobotanin and rutin were absent in the GESP, but rutin was found in the EFYT. However, carotenoid, cardiac glycoside, glycoside, phlobotanin, phenol, saponin, and tannin were absent in the EFYT.

**Table 2:** Qualitative detection of phytochemicals in GESP and EFYT.

Phytochemicals	GESP	EFYT		Phytochemicals	GESP	EFYT
Alkaloid	+	+		Phlobotanin	–	–
Albuminoids	+	+		Phenol	+	–
Anthracene	+	+		Quercetin	+	+
Anthraquinone	+	–		Reducing compounds	+	+
Betulinic acid	+	+		Rutin	–	+
Carotenoid	+	–		Saponin	+	–
Cardiac glycoside	+	–		β-sitosterol	+	+
Flavonoid	+	+		Steroid	+	+
Free anthraquinone	+	+		Sterols	+	+
Glucomannan	+	+		Stigmasterol	+	+
Glycoside	+	–		Tannin	+	–
Gums	+	+		Terpenoid	+	+
Lupeol	+	+				

GESP: Germinated endosperm of sugar palm; EFYT: Elephant foot yam tuber. Here, (+) and (−) indicates presence and absence respectively.

### 3.3 Antidiabetic effect of GESP, EFYT and their mixture on rats

**Fig. 1:**
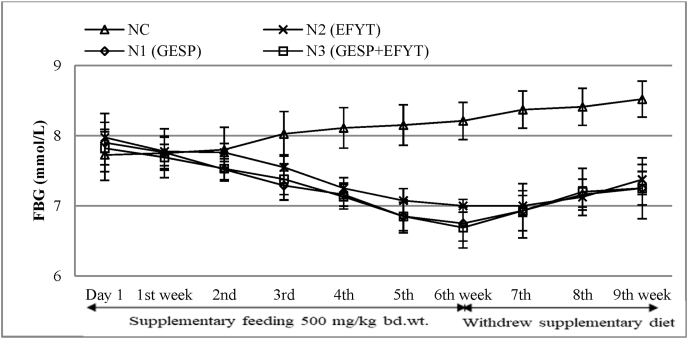
Comparative hypoglycemic scenario of EFYT and GESP on FBG level of nondiabetic rats.

FBG levels of both nondiabetic and diabetic rats were exposed in Table 3, and Table 4, respectively. No significant alterations in FBG levels of nondiabetic rats were found among the control (NC) and the three experimental groups, such as N1 (GESP), N2 (EFYT), and N3 (GESP + EFYT), as shown in Table 3. A slight increment of NC rats' FBG level was found with the progression of age from the initial day to 9th weeks (7.73–8.52 mmol/L). While N1, N2, and N3 had been shown a little bit lower FBG levels (7.9–6.75 mmol/L, 7.98 to 7.0 mmol/L, and 7.82 to 6.69 mmol/L respectively) when compared with 0 weeks to 6th weeks. The mild but non-significant hypoglycemic effects of GESP, EFYT, and the mixture (GESP & EFYT) were further shown by the onset of slow age-related increment of FBG levels after the withdrawal of supplementary therapeutic diet (Table 3). The overall scenario of FBG levels of nondiabetic rats was graphically presented in Fig. 1. As the supplementary feeding diet of GESP, EFYT, and their mixture (1:1) had no significant effects on the FBG levels of nondiabetic experimental rats; these therapeutic diets were presumed to be safe for consumption by normal rats.

**Table 3:** Effect of GESP and EFYT (500 mg/kg bd.wt./day) on FBG (mmol/L) level of non-diabetic rats (n = 6).

Weeks	NC	N1 (GESP)	N2 (EFYT)	N3 (GESP + EFYT)
0 week	7.73 ± 0.72	7.9 ± 0.72	7.98 ± 0.43	7.82 ± 0.47
1st week	7.75 ± 0.69	7.76 ± 0.4	7.78 ± 0.41	7.69 ± 0.37
2nd week	7.8 ± 0.64	7.52 ± 0.26	7.76 ± 0.26	7.53 ± 0.35
3rd week	8.03 ± 0.64	7.29 ± 0.36	7.55 ± 0.35	7.38 ± 0.38
4th week	8.11 ± 0.58	7.16 ± 0.29	7.25 ± 0.3	7.13 ± 0.35
5th week	8.15 ± 0.58	6.85 ± 0.35	7.08 ± 0.34	6.85 ± 0.41
6th week	8.21 ± 0.53	6.75 ± 0.44	7.0 ± 0.18	6.69 ± 0.58
After withdrew supplementary feeding				
7th week	8.37 ± 0.53	6.93 ± 0.49	7.0 ± 0.29	6.93 ± 0.67
8th week	8.41 ± 0.53	7.16 ± 0.38	7.13 ± 0.28	7.2 ± 0.75
9th week	8.52 ± 0.51	7.25 ± 0.41	7.37 ± 0.43	7.25 ± 0.87

GESP: Germinated endosperm of sugar palm; EFYT: Elephant foot yam tuber; NC: Non-diabetic control.

*p < 0.05; **p < 0.001; Mean ± standard deviation.

**Table 4:** Antidiabetic effect of GESP and EFYT (500 mg/kg bd.wt./day) on FBG (mmol/L) level of diabetic rats (n = 6).

Weeks	DC	D1 (GESP)	D2 (EFYT)	D3 (GESP + EFYT)
0 week	14.82 ± 0.6	15.35 ± 0.7	14.5 ± 0.9	15.4 ± 0.2
1st week	15.53 ± 1.1	14.2 ± 1.1	14.22 ± 0.6	14.48 ± 0.3
2nd week	16.67 ± 0.9	12.5^ba^±0.9	13.74^ba^±1.0	12.4^ba^±0.9
3rd week	19.58^aa^±1.2	10.6^aabb^±1.1	12.53^bb^±0.9	10.86^aabb^±0.6
4th week	21.89^ab^±1.1	9.54^abbb^±0.4	11.64^bb^±0.8	9.18^abbb^±0.8
5th week	23.55^ab^±1.3	8.93^abbb^±0.3	10.8^aabb^±0.4	7.92^abbb^±1.1
6th week	24.35^ab^±1.1	8.13^abbb^±0.8	10.01^aabb^±0.5	7.31^abbb^±0.6
After withdrew supplementary feeding				
7th week	26.03 ± 1.1	8.96 ± 0.2	10.98 ± 1.2	7.66 ± 0.4
8th week	27.64^ca^±1.1	9.53 ± 0.1	11.64 ± 0.4	8.04 ± 0.6
9th week	28.8^cb^±0.9	9.81 ± 0.2	12.45 ± 0.9	8.18 ± 0.7

GESP: Germinated endosperm of sugar palm; EFYT: Elephant foot yam tuber; DC: Diabetic control.

^a^p < 0.05.

^b^p < 0.001; Mean ± standard deviation.

^a^ indicates compare with initial day (0 week) in the same groups.

^b^ indicates compare with diabetic control group at the same week.

^c^ indicates compare with 6th week in the same group after omitting supplementary feeding.

The FBG level of both the diabetic control group (DC) and the three experimental groups viz. D1 (GESP), D2 (EFYT), and D3 (the mixture of GESP & EFYT) were shown in Table 4. FBG levels of DC rats were gradually increased throughout the study duration (up to 9th weeks), ranging from 14.82 to 24.35 mmol/L. At the same time, the FBG levels of D1, D2, and D3 were found to descending tendency until the supplementary feeding period (up to 6th weeks).

**Fig. 2:**
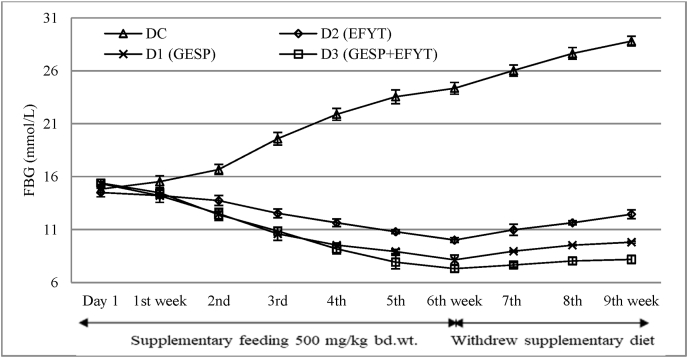
Comparative anti-hyperglycemic scenario of EFYT and GESP on FBG level of diabetic rats.

In the case of D1 (GESP), the FBG level was recorded 15.35 mmol/L at the beginning of the study, and exert reduced FBG levels below the lower limit of diabetes (<10 mmol/L) from the 4th weeks. However, the FBG levels of D1 were gradually and significantly (*P* < 0.001) reduced until the 6th weeks (8.13 mmol/L) when compared with DC at the same experimental time as well as with the initial day (0 weeks) of the same group. On the other hand, the FBG level of D2 (EFYT) was gradually reduced from the 2nd weeks, and significantly (*P* < 0.05) was noticed after the 5th weeks (14.5–10.01 mmol/L). But the supplementary feeding of EFYT unable to revert the diabetic rats into a nondiabetic compared to that of GESP by the end of the 6th week of the feeding trial. The reasons behind this difference may be due to the lower amount of fiber content as well as the absence of several phytochemicals such as carotenoid, cardiac glycoside, glycoside, phlobotanin, phenol, saponin & tannin in the EFYT than that of the GESP (Table 1, Table 2). The phenomenon was more clearly shown when the supplementary feeding of EFYT was withdrawn from the 7th to 9th weeks (Fig. 2). During this session, the FBG level of EFYT was gradually increased and remained within the range of diabetic (>10 mmol/L). However, a significant reduction (*P* < 0.05) of FBG levels of D2 were found after the 1st week and were highly significant (*P* < 0.001) from the 2nd to 6th weeks when compared with the DC rats.

The combined effect of GESP & EFYT (D3) was tested to see the efficacy of antidiabetic effects. Hopefully, the combined effect was more prominent than that of the distinct action of GESP and EFYT (as shown in Table 4).

The supplementary feeding diet for all experimental groups was withdrawn after the 7th weeks and continued for three weeks to see these diets' effectiveness. During this session, the FBG levels were not significantly altered compared to the FBG level of the corresponding 6th week. The increment of the FBG levels of DC rats was found to continue and reached 28.8 mmol/L by the end of the 9th week. While the FBG levels of all experimental (D1, D2, and D3) rats increased to some extent when compared to the FBG level of 6th week of the corresponding group but did not revert to the highest FBG levels of the same group (as shown in Table 4 and Fig. 2).

## 4 Discussion

The present study showed that GESP and EFYT were rich in carbohydrate, protein, fiber as well as dietary minerals viz. zinc, iron, copper, sodium, and potassium. Moreover, crude fat, free fatty acid, and non-protein nitrogen content were in a negligible amount. Besides, a remarkable amount of total carotenoids was present in the GESP.

A few studies have shown the nutritional values of EFYT and sugar palm fruit pulp, but no data on nutritional values of GESP is available till to date. According to Arup et al. [20], EFYT is rich with potassium (0.33%), phosphorus (0.17%), calcium (0.16%), and iron (0.003%). While Singh & Neeraj [21], reported starch (11–28%), sugar (0.7–1.7%), protein (0.8–2.60%), fat (0.07–0.40%), and minerals viz. calcium (0.13–0.25%), potassium (0.23–0.42%), phosphorus (0.12–0.25%), iron (1.97–5.56 mg), zinc (0.12–1.92 mg), manganese (0.19–0.65 mg), and soluble oxalate (6.65–18.50 mg). As nutritional values of fruits & vegetables differ among the varieties of the same species due to soil condition, environmental variation as well as genetic variations [22], the previous reports are accorded with the current study.

Secondary metabolites, like phytochemicals, have many functional roles in diabetes. Thus, the present study also investigated the qualitative test of twenty-five phytochemicals believed to have antidiabetogenic properties either directly or indirectly. Among them, twenty-three phytochemicals were found in the GESP, and only seventeen phytochemicals were present in the EFYT, as shown in Table 2. Study reports on *Amorphophallus* species had been revealed the presence of phytochemicals in corms viz. methanolic extract gave positive tests for steroids, flavonoids, alkaloids, sterols, terpenoids [23]; chloroform extract showed alkaloids, sterols, terpenoids [24]; petroleum ether gave alkaloids, steroids, sterol, terpenoids [21]; ethyl acetate and hexane of corm extracts gave alkaloid, and flavones [25]. Dey et al. [26] also added the absence of glycosides and saponins in the corm. Hence, the present result was consistent with the previous results.

As patients with hyperglycemia avoid GESP & EFYT as a myth of fearing that these foodstuffs may deteriorate blood sugar [8], the current study also had been designed on diabetic rats to clarify this phenomenon. The FBG level of the diabetic control group (DC) was gradually increased (14.82–24.35 mmol/L) throughout the study duration. At the same time, the FBG levels of D1 (GESP), D2 (EFYT), and D3 (GESP + EFYT) were found to descending tendency until the supplementary feeding period (up to 6th weeks).

The FBG level of D2 (EFYT) was gradually reduced from the 1st week, and significantly (*P* < 0.05) was noticed after the 4th week (14.5–10.01 mmol/L). But the supplementary feeding of EFYT unable to revert the diabetic rats into nondiabetic ones compared to that of GESP. This difference may be due to the lower amount of fiber content and the absence of several phytochemicals such as carotenoid, cardiac glycoside, and glycoside phlobotanin, phenol, saponin & tannin in the EFYT than that of the GESP (Table 2).

The combined effect of GESP & EFYT (D3) was more prominent than that of the distinct action of GESP and EFYT (as shown in Table 4). This may be due to having the highest number of phytochemicals (24 out of 25) and a higher amount of fiber & zinc. Current data strongly indicated that both nutritive and non-nutritive elements such as complex carbohydrate, fiber, minerals such as zinc, and important phytochemicals could be beneficial for diabetic patients [27], probably by regenerating the damaged pancreatic β-cells [28,29], facilitating glucose uptake by the tissues [30,31], and inhibition of the α-glucosidase as well as α-amylase enzyme activity [32]. Among the antidiabetic nutrients-fiber and saponin lower the intestinal glucose uptake [33,34]; zinc, glycosides, saponin, lupeol, etc. regenerate β-cells [[35], [36], [37]]; betulinic acid, stigmasterol, β-sitosterol decrease humoral regeneration of glucose via inhibition of α-glucosidase & α-amylase [38]; anthraquinone sensitize insulin receptors [39] as well as those having antioxidant properties play the role to prevent β-cell destruction [40,41].

The GESP and EFYT exert an antidiabetic effect, probably via pancreatic β-cell regeneration. The probability was suggested by the successive three weeks (7th to 9th weeks) withdrawal of the supplementary feeding diet. During this session, the FBG levels were not significantly altered compared to the FBG level of the corresponding 6th week. The increment of DC rats' FBG levels was found to continue and reached 28.8 mmol/L by the end of the 9th week. While the FBG levels of all experimental (D1, D2, and D3) rats increased to some extent compared to the FBG level of the 6th week of the corresponding group but did not revert to the highest FBG levels of the same group. These results indicated that the therapeutic administration of GESP, EFYT, and their mixture might repair the alloxan damaged pancreatic β-cells differently with the variations of their composition. A combination of GESP & EFYT feeding has the highest damage repair effects. GESP administration has the second-highest impact and is followed by the EFYT.

## 5 Conclusion

GESP and EFYT are popularly consumed by the peoples of Bangladesh and other neighboring countries. Although a few groups have studied the nutritional composition of different varieties of EFYT, the nutrient composition of GESP is unknown to date. Current investigation showed that GESP and EFYT are not harmful to diabetic rats; instead, they exert hypoglycemic effects by their contents, especially phytochemicals, fiber, sodium, potassium, copper, and zinc. The combination of GESP & EFYT showed the most antidiabetic effects followed by GESP as both contain the maximum number of antidiabetogenic phytochemicals as well as nutrients. Thus both of these foodstuffs should be considered suitable for the dietary management of type 2 diabetic patients. Further study should be carried out to investigate the mechanism of antidiabetic effects as well as purify the antidiabetic components from these foodstuffs.

## Author contributions

**Shaikh Shahinur Rahman:** Conceptualization, Formal analysis, Investigation, Data curation, Writing- original draft. **Hussain Mohammad Salauddin:** Formal analysis. **Mahfuzur Rahman:** Data curation. **Mir Mohammad Muhsin:** Formal analysis. **Shakh MA Rouf:** Conceptualization, Methodology, Investigation, Data curation, Writing- review & editing.

## Declaration of competing interest

Authors declare no conflict of interest in this manuscript.
